# Thermal-mechanical coupling failure analysis and optimization of an exhaust manifold

**DOI:** 10.1016/j.mex.2026.103877

**Published:** 2026-03-23

**Authors:** Long Haiqiang, Zhu Hao, Shao Jian

**Affiliations:** a Chongqing City Management College, Chongqing 401331, China; b Failure Mechanics & Engineering Disaster Prevention and Mitigation (Key Laboratory of Sichuan Province), Department of Mechanics and Engineering Science, Sichuan University, Chengdu 610065, China; c College of Mechanical and Vehicle Engineering, Chongqing University, Chongqing, 400044, China

**Keywords:** Exhaust manifold, Finite element method, Thermal-mechanical coupling, Optimization

## Abstract

Exhaust manifold is an important component of the engine system, and the failure of manifold can cause faults such as reducing power and increasing noise. This study adopted finite element simulation and experimental methods such as high-temperature tensile testing, metallographic analysis to investigate stress characteristics under mechanical and thermal-mechanical coupling loads. The Mises stress of the exhaust manifold under thermal-mechanical coupling load reaches 150MPa, and it closes to yield stress of material at the temperature of 600°C. High temperature and installation constraints restrict the expansion of the exhaust manifold and results in significant high stress and stress concentration. The thermal-mechanical load is the main reason leading to the failure of the exhaust manifold. Constraints optimization is applied to redesign the structure and installation constraints. It is an effectively method to reduce structural stress and improve the fatigue resistance of the manifold.

• Geometric scanning method is applied to analyze the manufacture deviation of the exhaust manifold.
• Material failure analysis and material testing with different temperature are applied in the study.
• Constraint conditions and high temperature mainly effect the stress of the exhaust manifold.

## Graphical abstract

**Figure:**
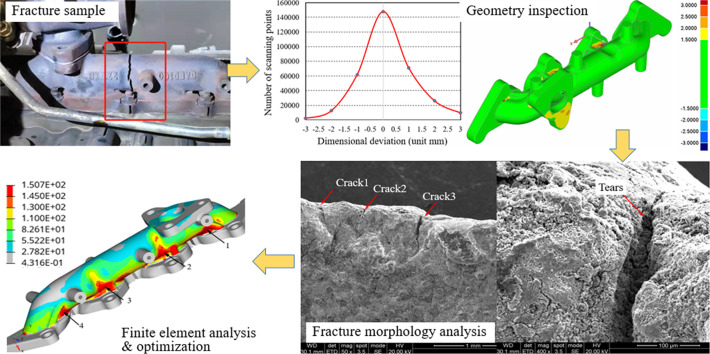

## Specifications table

**Table:** 

Subject area	Engineering
**More specific subject area**	*Failure analysis*
**Name of your method**	*Mechanical –thermal coupling failure analysis*
**Name and reference of original method**	*/*
**Resource availability**	*Finite element analysis software, ABAQUS v6.14-1*

## Background

**Fig. 1:**
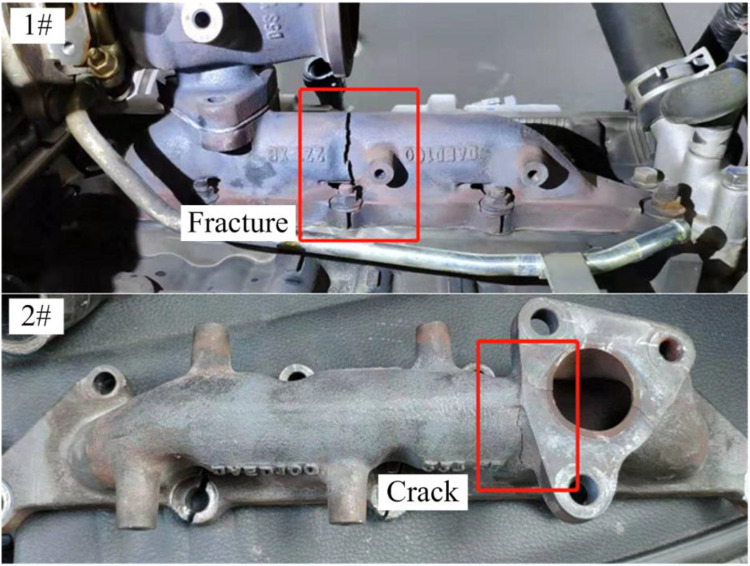
Failure of the exhaust manifold. Sample 1# fractured and sample 2# cracked during the proving ground testing at the mileage around 30000 km.

Exhaust system is an essential component in a gasoline engine, which controls the flow of the burnt fuel out of the engine cylinders. The main function of the exhaust manifold system is to collect and discharge the exhaust gases generated during the engine combustion process from multiple cylinders [1,2]. The exhaust manifold is an important component of the engine exhaust system, and its working under harsh conditions. The continuous improvement of performance causes the exhaust temperature of exhaust airflow reaching up to 900 °Cor even higher [3,4]. Proving ground test is required in the development of new vehicle products. The new car development proving ground test requires that the exhaust manifold can withstand a 40000 km road test. While, during the proving ground test of a new passenger car development, two of testing vehicles got cracking and failure on component of exhaust manifold. Fig. 1 shows the proving ground testing checking results. The exhaust manifolds get broken or cracked at the mileages ranging from 30000 km to 35000 km. Sample 1# experienced fracture failure at the test mileages of 35000 km, and sample 2# exhibited obvious cracks at the test mileages of 30000 km, respectively.

The fracture failure of the exhaust manifold will further leads to a sharp decrease in exhaust pressure and power, and increase in noise [[5], [6], [7], [8]]. The working loads of exhaust manifold including vibration loads from engine and road excitation, as well as high-temperature load from exhaust airflow [9,10]. Studying the causes of exhaust manifold failure is a key factor in improving product resistance to failure. In order to analyze the causes of the fracture and failure on the exhaust manifold, material failure analysis and nonlinear finite element analysis method are applied in this study. The effect of mechanical load, thermal load and thermal-mechanical coupling load on the manifold are carried out by finite element analysis method. Based on the comprehensive analysis results, constraints optimization and design are applied to enhance the manifold`s resistance to failure.

### Method details

The exhaust manifold is subjected to various loads, and the reasons for its failure may include structural design, material properties, working loads, and other factors. To study the causes and effects of exhaust manifold failure, this article conducts research on exhaust manifold manufacturing deviations, fracture morphology characteristics, material properties, working loads, and constraint boundary conditions. The method detail is organized as follows. The geometry inspection is presented in the next section. Fracture morphology and material properties are shown following geometry inspection. Finally, the finite element analysis is applied to study the effect of thermal load and constraint condition on the exhaust manifold.

### Geometry inspection

**Fig. 2:**
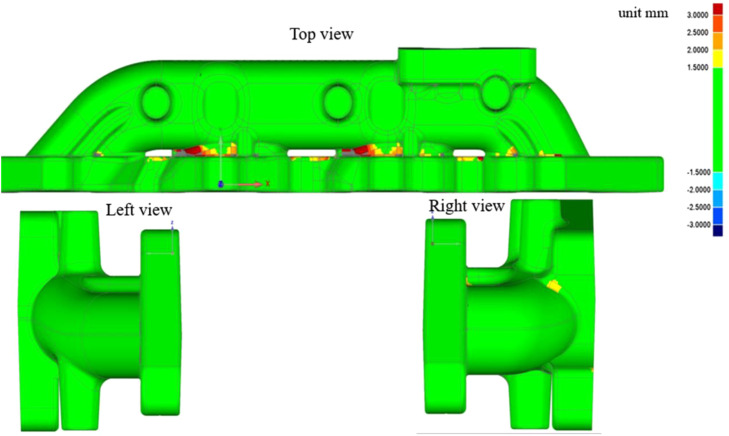
Geometric scanning and deviation view of different direction.

Dimensional deviation of exhaust manifold in the manufacturing process may leads to installation difficulties and even affects the structural stress and utilization. The comparison of 3D scanning on the surface of the exhaust part can check the manufacturing dimension deviation. The geometry scanning is applied from six directions, and Fig. 2 shows the scanning view of top, left and right. The surface dimension deviation of the exhaust manifold is represented in the form of a cloud map, where green indicates the area with smaller size deviation and red indicates the area with the largest size deviation. The maximum dimension deviation is less than 3 mm. The surface scanning results show that the dimension deviation. The areas with large dimensional deviations are mainly concentrated in the cast fillet of the outer surface of the middle exhaust ducts.

**Fig. 3:**
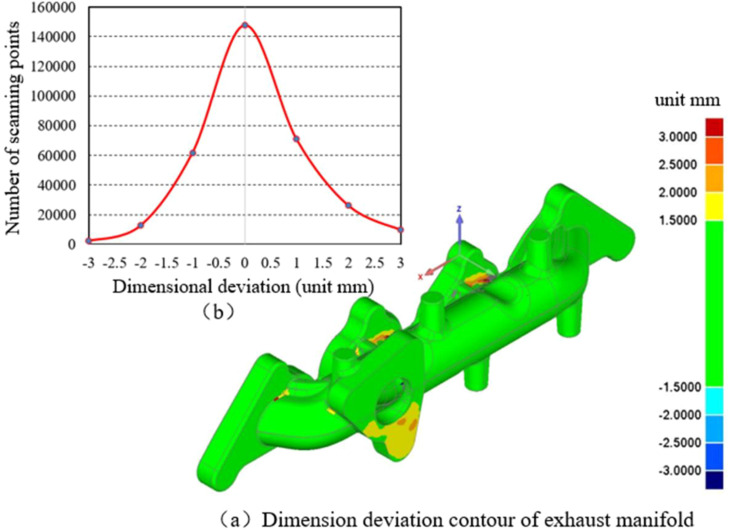
Geometric scanning and deviation analysis of the exhaust manifold.

Further comparisons between manufacturing 3D scanning data and geometrical design are presented in Fig. 3. A total of 330 thousands scanning points are used to form the inner and outer surfaces of the manufactured manifold. The isometric view of the geometric scanning results and deviation analysis are shown in Fig. 3(a) and (b), respectively. Fig. (a) shows the isometric view of geometric comparison analysis based on the scanning of inner and outer surfaces of the exhaust manifold. The manufacturing deviation analysis result shows that the maximum deviation is less than 3 mm. The regions with significant manufacturing dimensional deviations are mainly concentrated at the manufacturing fillets, and the dimensional deviations in these regions do not affect the installation of the exhaust manifold. A statistical analysis of manufacturing deviations is shown in Fig. (b), and the statistical result indicates that the manufacturing deviation of most inspection points is less than 1mm, accounting for 85 %. The geometric inspection result indicates that the manufacturing of the exhaust manifold meets its design requirements, and the manufacturing deviation does not affect the installation and it is the cause of failure.

### Failure analysis

**Fig. 4:**
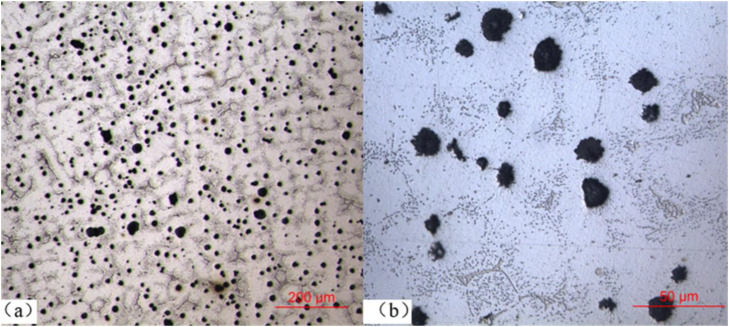
Metallographic analysis, Fig. (a) shows a 100x magnification and Fig. (b) shows a 400x magnification.

To analyze the issue of exhaust manifold failure from the microstructure, a metallographic microscope was used to analyze the metallographic composition of the central and surface of the fractured sample 1#. The metallographic analysis result shows that both of the central and surface of the fractured sample have the same metallographic compositions. Fig. 4(a) and (b) show the metallographic micrographs of the central fracture surface, where, (a) is at magnification factor of 100 and (b) is at magnification factor of 400, respectively. The metallographic analysis of the fracture surface shows that the spheroidization rate of graphite exceeds 90 %, and the metallographic compositions are mainly composed of austenite and carbides, and also composed of a few alloy phases and dendrites.

**Fig. 5:**
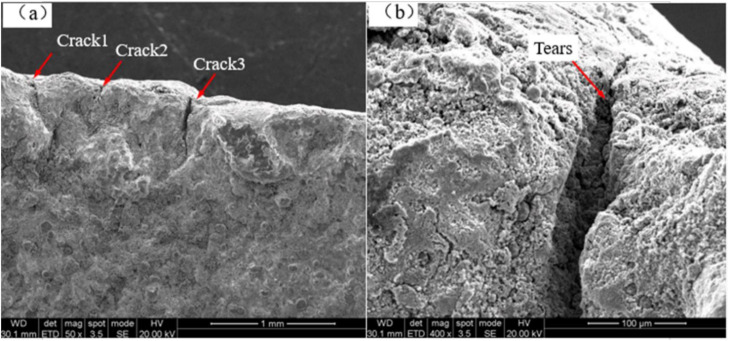
Fracture morphology, Fig. (a) and (b) show the 50x and 400x of the facture initiation respectively.

Scanning electron microscopy was further used to analyze the morphology characteristics of fracture surfaces as shown in Fig. 5(a) and (b). The fracture scanning photo shows that the initiation zone of the failure specimen is located on the inner surface of the exhaust manifold. Fig. 5(a) shows a 50x analysis result of the initiation and propagation zones of the fracture surface. The initiation zone obviously exhibits multi-point initiation characteristic, and intergranular traces are visible in the crack propagation area. Fig. 5(b) presents a 400x photo of the fracture initiation zone, and it further reveals the tearing marks and visible cracking on the inner surface of the exhaust manifold. These characteristics reveal that the exhaust manifold failure shows typical features of thermal fatigue failure. The fracture morphology results can be concluded that the fracture of the exhaust manifold belongs to thermal fatigue failure.

The exhaust manifold working in a harsh environment, and high temperature usually leads to a decrease in material mechanical properties, thereby accelerating the failure of exhaust manifold [11]. Material performance testing considering the influence of temperature is applied to study the effect of high temperature on exhaust manifold material. This article refers to the ASTM E21-2020 experimental standard and uses a tensile testing machine with heating function to test influence of temperature on the mechanical properties. This standard is applied for testing of high-temperature properties of metallic materials. It specifies the requirements for heating devices and temperature systems, clamping devices, test samples, test methods, equipment accuracy, and deviations. Tensile test is the most frequently used method for determining material properties under axial tensile load. The tensile test data can be used to determine the material's tensile performance indicators such as elastic limit, elongation, elastic modulus, yield strength, and tensile strength and so on. Meanwhile, the tensile test conducted at high temperature can also obtain the effect of temperature on the tensile properties of the testing materials. The exhaust manifold is affected by the high temperature of the exhaust airflow, and the outer surface temperature of the manifold can reach over 500°C. In order to investigate the effect of temperature on exhaust manifold materials, tensile tests were conducted on the samples at room temperature and the high temperature reaches 700°C.

**Fig. 6:**
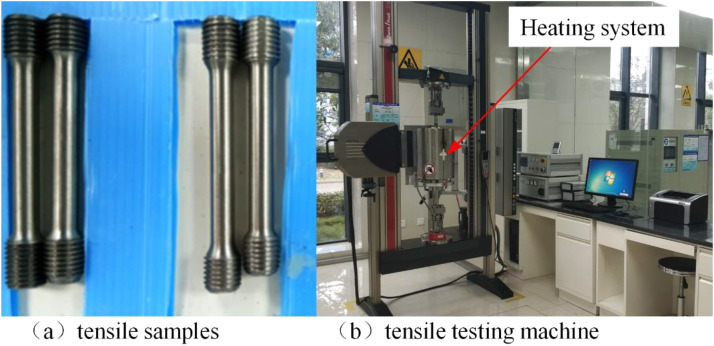
Tensile samples and test, (a) shows the tensile samples and (b) shows the tensile machine with heating system.

**Fig. 7:**
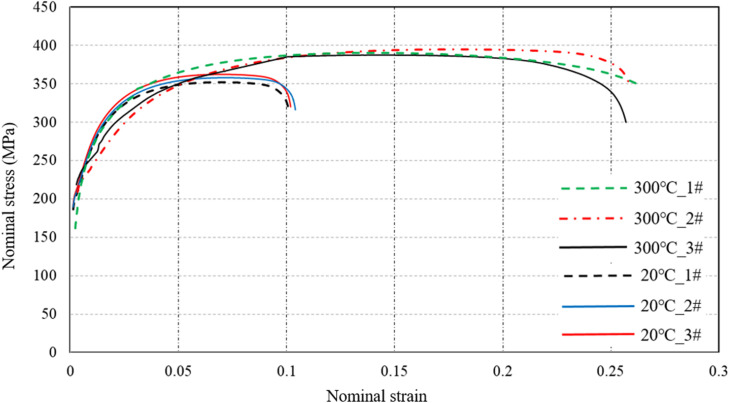
Nominal stress vs strain of test samples at 300 °C and 20°C.

Fig. 6 shows the test machine and specimens of the tensile test according to ASTM E21-2020 standard. Test specimens are made by the material named QTAN_i_35S_i_5C_r_2 according to the exhaust manifold manufacture. A total of 15 specimens are used in the tensile test and some of the test specimens are shown in Fig. 6(a). The diameter of the test sample is 10 mm, the length of the tensile marking region is 100 mm, and the surface machining roughness is Ra=0.8μm. Threads are produced at both ends of the specimens to ensure clamping requirements. A universal tensile testing machine with heating function is applied for tensile test as shown in Fig. 6(b). The tensile test is performed under quasi-static tensile conditions, and set the stretching speed to 4 mm/min. Considering the working temperature of the exhaust manifold, the tensile test of the sample is mainly conducted under 20 °C and high temperature conditions of 300-700°C. Fig. 7 shows the typical nominal stress-strain curves of tensile specimens at 20 °C and 300°C. The nominal stress-strain shows the maximum stress increased slightly, while the strain increased by 150 %.

Nominal stress and strain experimental curves show the mechanical properties of the individual testing sample. Date processing is applied for the samples to reveal the mechanical properties of materials under different temperature conditions. According to the averaged processing, the yield strength, tensile strength, elongation and ultimate tensile strength parameters of testing samples under different temperatures are listed in Table 1. The statistical results show that compared with 20°C, the elastic modulus, yield strength, and tensile strength of the material are comparable at 300°C, while the elongation rate has been significantly improved. When the temperature is above 500°C, the elastic modulus, yield strength, and tensile strength of the material show a significant downward trend. The decrease in elastic modulus is significant. This indicates that high temperature significantly softens the material and reduces its load-bearing capacity.

**Table 1:** Material parameters.

Temperature [°C]	Young modulus E [×10^5^ MPa]	Yield stress YS [MPa]	Elongation [%]	Ultimate tensile strength UTS [MPa]
20	131	182	10.2	368
300	133	175	25.6	385
500	112	172	27.3	375
600	95	161	30.2	315
700	80	152	35.1	246

### Mechanical-thermal coupled analysis

The geometry inspection, fracture morphology and material properties test are applied to study the failure characteristics, and the result concluded that the exhaust manifold belongs to thermal fatigue failure. Stress and distribution characteristic of the exhaust manifold is the fundamental causes of its failure. While, these experimental work are not convenient for directly studying the stress distribution and failure effect of exhaust manifolds. The finite element analysis method is an effective approach for studying structural stress characteristics and is widely used to investigate various mechanical failure problems. The finite element analysis process involves modeling, performing loading and simulation calculations [12,13]. This article adopts finite element analysis method to study the structural stress characteristics considering mechanical and thermal coupled load, further information and working process is executed as follows.

**Fig. 8:**
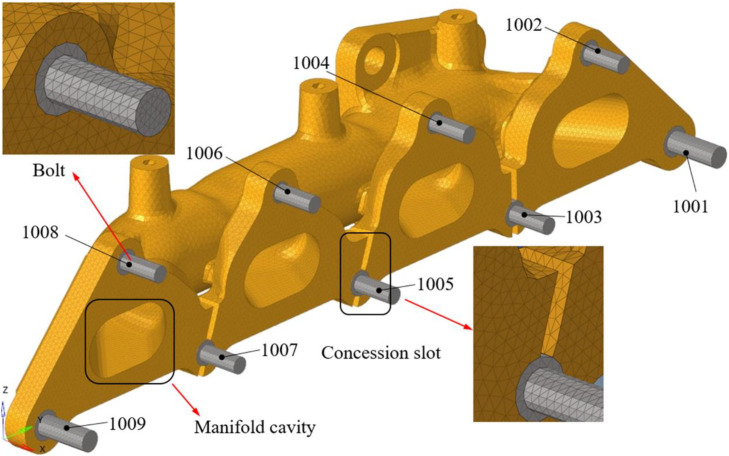
Finite element model of manifold.

The software ABAQUS is applied in the stress analysis and the exhaust manifold is modeled with solid elements. The manifold is subjected to multiple loads such as mechanical vibration loads, gravity field loads, and high-temperature thermal loads during operation process. C3D4H (4-node linear tetrahedron and hybrid element) element is used in the simulation modeling. The C3D4H element can simulate the coupling of structural loads and thermal loads. The element size is an important factor effect the model scale and computation time. The minimum casting fillet of the exhaust manifold is 4mm. In order to balance simulation accuracy and computational efficiency, the averaged element length of this model is defined as 2 mm. Fig. 8 shows the finite element model of the exhaust manifold, which is modeled with 148445 tetrahedral elements. The manifold system model contains manifold and fixed bolts which are used to connect the exhaust manifold and engine. The fixed bolts are numbered form 1001 to 1009, and they are used to apply constraint conditions in the analysis process. The connections between fixed bolts and manifold mounting holes are modeled by using common nodes. The casting fillets and manifold cavities are modeled consistent with geometric design. Triangular elements is used to establish closed faces along the inner and outer surfaces of the exhaust manifold, and then tetrahedral elements are generated in the closed cavity.

**Fig. 9:**
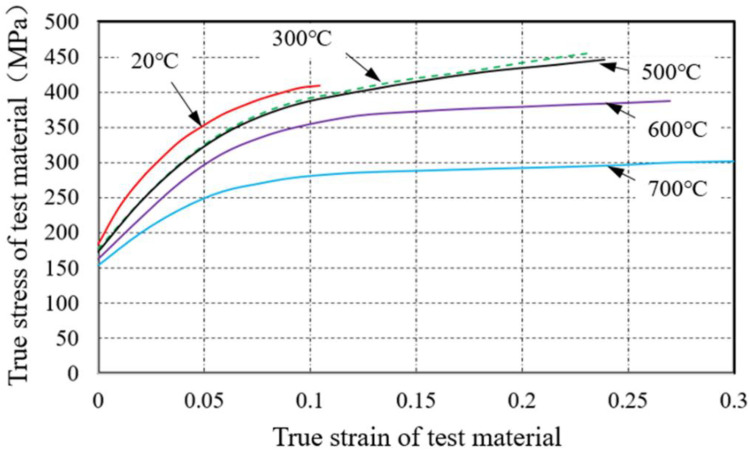
The true stress-strain curves of the material used in the finite element analysis.

ABAQUS software is suitable for solving linear and nonlinear problems. In order to simulate the stress characteristics of exhaust manifolds more accurate, this paper applies nonlinear analysis methods to simulate the working stress and stress distribution of exhaust manifolds. True stress vs true strain properties of the material is necessary in the nonlinear analysis of ABAQUS. According to the working temperature and material properties tests of the exhaust manifold, the true stress-strain curves of the materials corresponding to different temperatures are shown in Fig. 9. In ABAQUS software, the true stress-strain curves of material starting from the yield point and ending at the point of rupture. As show in Fig. 9, five true stress-strain curves are defined to present the material properties at different temperature. The true stress-strain curve is a continuously increasing stress curve, and the increase in temperature enhances the true strain of the material.

**Fig. 10:**
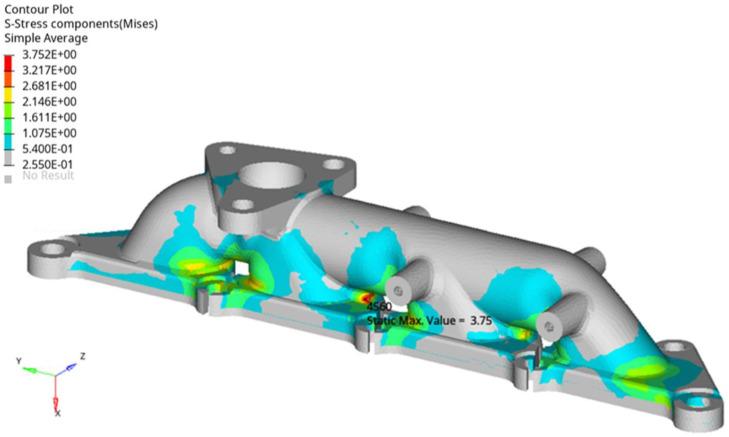
Stress of manifold under mechanical loads (MPa).

The mechanical load cases mainly consider the structural stress caused by the working excitation of the exhaust manifold. The exhaust manifold is fixed on the engine block by the bolts, therefore, the bolts numbered 1001 to 1009 are constrained in the finite element simulation. The exhaust manifold loads including mechanical loads and thermal loads, and mechanical loads is applied to simulate the stress in the first step. According to the product design load specification, the mechanical loads analysis is applied with the static loads composed with x_8G, y_10G, and z_15G, and all the fixed bolts are constrained the moving freedoms along the coordinate axes. Fig. 10 shows the maximum von-Mises stress is 3.75 MPa, and located in the outer surface of the manifold. Material tensile test shows the yield stress of the material is about 150 MPa the temperature of 700°C, meanwhile, the maximum stress under mechanical load is significantly less than the yield strength. The mechanical load analysis result shows that, the mechanical load is not the main reason which causes the failure of the exhaust manifold. In the further studies, thermal load and effect constraint conditions are considered in the finite element analysis.

**Fig. 11:**
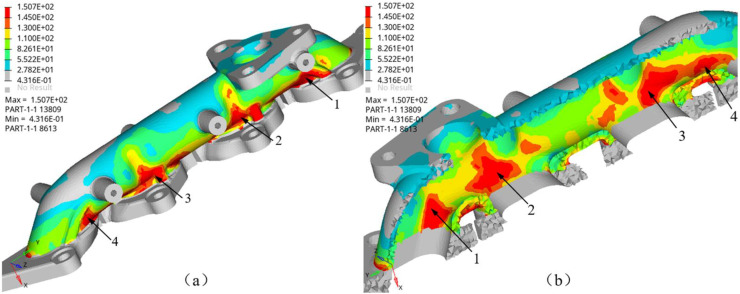
Thermal-mechanical coupling analysis, (a) shows the stress at the outer face and (b) shows the stress at the inner face (MPa).

Exhaust manifold temperature test shows that the engine output power affects the exhaust manifold temperature. When the engine operates stably at 4000 revolutions per minute, the temperature of the exhaust manifold outer surface is around 550°C. Combining temperature testing and heat conduction analysis, the temperature on the inner surface of the manifold is approximately 650°C. Mechanical load coupled with thermal loads of 650 °C and 550 °C are applied at the inner and outer surfaces of the manifold respectively. Similarly, all the connecting bolts are constrained the moving freedoms. The thermal and structural coupling stress of the exhaust manifold is calculated using a sequential coupling method by ABAQUS software. The thermal mechanical coupling of the exhaust manifold is divided into two steps. The first step is to constrain the bolt connection position and apply mechanical loads to calculate the structural stress distribution. Then, the followed step maintains the mechanical loads and adding the thermal loads. Fig. 11 shows the overall stress distribution of thermal mechanical coupling loads in the exhaust manifold. Among them, Fig. 11(a) describes the stress distribution on the outer surface of each flow channel, and Fig. 11(b) shows the stress on the inner surface of the exhaust manifold. The maximum stress is 150.7 MPa, and located at the inner face of channel 1of the manifold. The maximum stress is close to the yield strength of the exhaust manifold material at 700°C. Meanwhile, high stresses also distribute on the outer surfaces of channels 1, 2 and 3. The high stress located coincident with fracture or crack positions. It indicates that mechanical and thermal coupled load causes high stress and stress concentration, and that is the main factors leading to exhaust manifold failure.

### Effect of constraint conditions

Mechanical structures often experience thermal expansion due to temperature increase. If the structure can expand freely without restriction, it will not generate structural stress. In fact, installing constraints is a necessary condition for the operation of mechanical structures. While, the constraint boundary of component installation restricts the expansion of the structure, resulting in thermal stress in the structure. Therefore, studying the influence of installation constraints on structural thermal stress is important for reducing structural thermal stress and optimizing structural design. Based on the mechanical thermal coupled finite element analysis method, this work studies the effect of constraints on stress characteristic.

**Fig. 12:**
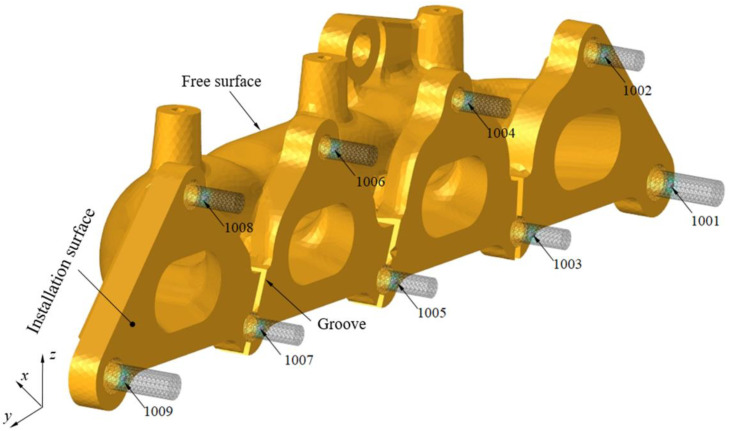
Fixed points and constraint analysis.

Fig. 12 shows the manifold can potentially fix to the engine cylinder through 9 bolt holes numbered from 1001 to 1009. The direction of the coordinate system in the Fig. is consistent with the coordinate system of the vehicle. The installation characteristics of the exhaust manifold indicate that the expansion in the x-direction of the installation surface is constrained by surface contact, and the unconstrained surfaces can expand freely. The size of the exhaust manifold in the y-direction is significantly greater than that in other two directions. Therefore, the main reason for the high stress of the exhaust manifold is due to the constraint of bolts on the expansion in the y-direction. The constraint position, displacement constraint direction, and local stiffness of the structure affect the thermal expansion and stress of the exhaust manifold. To reduce thermal expansion stress, the exhaust manifold is designed with grooves to reduce expansion constraints and lower constraint stiffness.

**Fig. 13:**
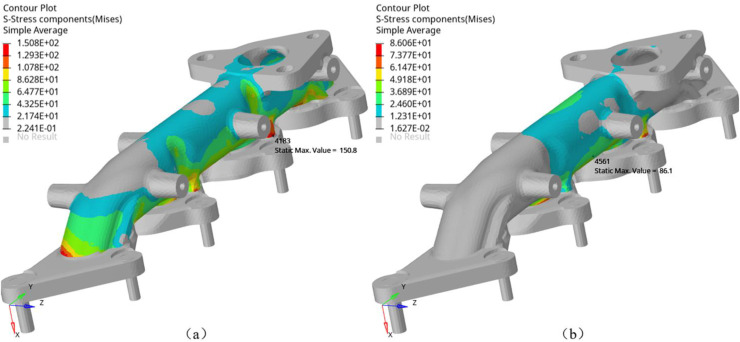
Fixed points and constraint analysis.

Finite element simulation method is applied to study the influence of constraint positions and direction on the thermal stress of the manifold. In finite element analysis, some of the fixed bolt can constraint all the translational freedom along x, y, z axis, and some of the fixed point can constraint translational freedom along x and z axis. Fig. 13 compares the stress and distribution at different constraint positions under the temperature of 650°C. Fig. 13a constrains all the translational freedom of bolts numbered 1001 to 1009, while Fig. 13b constrains the middle three bolts numbered 1004 to 1006. The maximum Mises stress decreases from 150.8 MPa to 86.1 MPa, and the stress is mainly distributed in the structure between the constraint points. The comparison results indicate that the constraint conditions significantly affect the maximum stress and stress distribution of the exhaust manifold structure. Constraints restrict thermal expansion of the exhaust manifold, which leads the stress increases. Therefore, releasing the constraint freedom or reducing the stiffness of the exhaust manifold along its main expansion direction can help reduce structural stress.

### Method validation

The aforementioned results indicate that the constraints in the x and z directions of the exhaust manifold are primarily due to installation requirements, while the constraint in the y direction is the main factor affecting thermal expansion stress. Base on this method, further detail analysis is applied to carry out better constraint position, constraint points and directions. A series analysis with constraints are applied to study exhaust manifold stress. The results show that constrained all the three-dimensional translational freedom at the bolts numbered 1002,1005 and 1008, and the bolts numbered 1001, 1003, 1004, 1006, 1007 and 1009 constrained the x and z-direction translational freedom can achieve more reasonable installation and stress distribution results. The maximum Von-Miese stress on the exhaust manifold is 145.2 MPa, and the stress distribution is relatively uniform throughout the exhaust manifold. The exhaust manifold has 3 fully constrained points and 6 constrained points in the x and z directions to ensure the installation requirements.

## Limitations

This article mainly analyzes the causes and influencing factors of exhaust manifold failure. The proposed workflow is intended for rapid failure diagnosis and structural design guidance under thermo-mechanical loading, rather than for fatigue life or creep life prediction. Design optimization products should further increase experimental verification. This article has not yet conducted in-depth research on material creep, thermal fatigue simulation, and corresponding experiments. Meanwhile, time-dependent creep and cyclic fatigue effects are not explicitly modeled in this study and are recommended for future work focused on life prediction.

## Ethics statements

This work does not involve human subjects, animal experiments, and data collected form social media platforms.

## Related research article

Failure and thermal-mechanical coupling analysis of exhaust manifold of a gasoline engine, Structures

## For a published article

Failure and thermal-mechanical coupling analysis of exhaust manifold of a gasoline engine, Structures

## CRediT author statement

The outlines of the Contributor Roles Taxonomy (CRediT) for this research project is presented as follows.

Long Haiqiang worked as the roles of Formal analysis, Methodology, Validation, Writing original draft, review & editing.

Zhu Hao worked as the roles of Methodology, Visualization, review & editing.

Shao Jian worked as the roles Software, Resources, Visualization & editing.

## Supplementary material *and/or* additional information [OPTIONAL]

No supplementary material

## Declaration of competing interest

The authors declare that they have no known competing financial interests or personal relationships that could have appeared to influence the work reported in this paper.

## Data Availability

Data will be made available on request.

## References

[1] B M N A, A H K, D M B C (2017). Combustion and emission characteristics of DI diesel engine fuelled by ethanol injected into the exhaust manifold-Science Direct. Fuel Process. Technol..

[2] Benoit A., Maitournam M H, Remy L. (2012). Cyclic behaviour of structures under thermo mechanical loadings: application to exhaust manifolds. Int. J. Fatigue.

[3] Yan Z., Zhien L., Wang X. (2014). Cracking failure analysis and optimization on exhaust manifold of engine with CFD-FEA coupling. Proc. R. Soc. A.

[4] Witek Lucjan. (2016). Failure and thermo-mechanical stress analysis of the exhaust valve of diesel engine. Eng. Fail. Anal..

[5] Salehnejad M A, Mohammadi A., Rezaei M. (2019). Cracking failure analysis of an engine exhaust manifold at high temperatures based on critical fracture toughness and FE simulation approach. Eng. Fract. Mech..

[6] Li Y., Liu J., Huang W. (2023). Failure analysis of a diesel engine exhaust manifold. Int. J. Met..

[7] Habashneh M., Rad M M (2023). Reliability based topology optimization of thermoelastic structures using bi-directional evolutionary structural optimization method. Int. J. Mech. Mater. Des..

[8] Qiu S., Yuan Z C, Fan R X (2019). Effects of exhaust manifold with different structures on sound order distribution in exhaust system of four-cylinder engine. Appl. Acoust..

[9] Lekakh S N, Johnson C., Bofah A. (2021). Correction to: improving high-temperature performance of high si-alloyed ductile iron by altering additions. Springer Int. Publ..

[10] Royale A., Simic M., Lappas P. (2021). Engine exhaust manifold with thermoelectric generator unit. Int. J. Engine Res..

[11] Yuan B., Ye H., Li J. (2023). Topology optimization of geometrically nonlinear structures under thermal-mechanical coupling. Acta Mech. Solida Sin..

[12] Amaechi C.V., Chesterton C., Butler H.O. (2022). Finite element modelling on the mechanical behaviour of marine bonded composite hose (MBCH) under burst and collapse. J. Mar. Sci. Eng..

[13] Gosztola,et D. (2024). Plastic limited numerical modelling on contact friction effects of steel–concrete connection for composite bridges. Buildings.

